# Microbiological and Clinical Aspects of Cervicofacial *Actinomyces* Infections: An Overview

**DOI:** 10.3390/dj7030085

**Published:** 2019-09-01

**Authors:** Márió Gajdács, Edit Urbán, Gabriella Terhes

**Affiliations:** 1 Department of Pharmacodynamics and Biopharmacy, Faculty of Pharmacy, University of Szeged, 6720 Szeged, Eötvös utca 6., Hungary; 2 Institute of Clinical Microbiology, Faculty of Medicine, University of Szeged, 6725 Szeged, Semmelweis utca 6., Hungary; 3 Department of Public Health, Faculty of Medicine, University of Szeged, 6720 Szeged, Dóm tér 10., Hungary

**Keywords:** *Actinomyces*, actinomycosis, cervicofacial infection

## Abstract

Similarly to other non-spore-forming Gram-positive anaerobes, members of the *Actinomyces* genus are important saprophytic constituents of the normal microbiota of humans. *Actinomyces* infections are considered to be rare, with cervicofacial infections (also known as ‘lumpy jaw syndrome’) being the most prevalent type in the clinical practice. Actinomycoses are characterized by a slowly progressing (indolent) infection, with non-specific symptoms, and additionally, the clinical presentation of the signs/symptoms can mimic other pathologies, such as solid tumors, active *Mycobacterium tuberculosis* infections, nocardiosis, fungal infections, infarctions, and so on. The clinical diagnosis of actinomycosis may be difficult due to its non-specific symptoms and the fastidious, slow-growing nature of the pathogens, requiring an anaerobic atmosphere for primary isolation. Based on 111 references, the aim of this review is to summarize current advances regarding the clinical features, diagnostics, and therapy of cervicofacial *Actinomyces* infections and act as a paper for dentistry specialists, other physicians, and clinical microbiologists.

## 1. Introduction, Taxonomy

*Actinomyces species* (originating from the greek words *aktinos* (ray) and *mykes* (fungus), corresponding to the radial arrangement of the bacterial filaments) are members of anaerobic, non-spore-forming Gram-positive rods [1,2]. Taxonomically, the genus *Actinomyces* is part of the *Actinomycetales* order of the Actinobacteria phylum (characterized by high guanine-cytosine (G+C) content in their genome, 55–68% in case of the *Actinomyces* genus) [3,4]. The *Actinomycetales* order includes other clinically important genera, such as *Actinobaculum*, *Actinomadura*, *Corynebacterium*, *Cutibacterium* (previously *Propionibacterium*), *Frankia*, *Gardnerella*, *Mobiluncus*, *Nocardia*, and *Varibaculum*. The genera *Actinobaculum*, *Actinomyces*, *Mobiluncus*, and *Varibaculum* are the most similar phenotypically, as they share several characteristics (a highly pleomorphic morphology ranging from branching rods to coccobacilli, various levels of aerotolerance, and non-acid fast staining, which is an important differentiating factor from *Nocardia* species) distinct from the other members of the order [5,6]. Nevertheless, several genera, including *Actinomyces*, *Frankia, Nocardia*, and *Streptomyces* are sometimes (incorrectly) referred to as the “*actinomycetes*” group in clinical practice, due to their similar radiating or branching morphologies (resembling hyphae) and the possession of reproductive asexual spores, typical for filamentous fungi [7,8,9]. The genus *Actinomyces* currently holds 49 species, of which more, than 26 species have been implicated in human clinical infections (Table 1), however this number may be a reported estimation only, as these pathogens were frequently misidentified before the introduction of current diagnostic technologies (e.g., DNA-DNA hybridization, polymerase chain reaction (PCR), matrix-assisted laser desorption/ionization time-of-flight mass spectrometry (MALDI-TOF MS), and next-generation sequencing (NGS)) [1,3,5,7,8,10,11].

Similarly to other non-spore-forming Gram-positive anaerobes, members of the *Actinomyces* genus are important saprophytic constituents of the normal microbiota of animals and humans [2,12,13]. These microorganisms are predominantly found in the human oropharynx, and they are abundantly present in gingival crevices, periodontal pockets, tonsillar crypts, as well as on carious teeth and in dental plaques [1,5,8,11,14]. In fact, ~30% of edentulous infants are colonized at the age of 2 months, while at the age of 12 months, >90% of children harbored these microorganisms in their oral cavities; in these studies, *A. odontolyticus* was found to be the predominant member of the genus, while interestingly *A. israelii* (the major etiological agent in human infections) was uncommonly isolated [1,5,8,11,14]. Among the results of the National Institutes of Health (NIH) Human Microbiome Project (HMP), other species, such as *A. georgiae*, *A. gerencseriae*, *A. israelii*, *A. meyeri*, *A. naeslundii*, *A. odontolyticus*, *A. oricola*, and *A. radicidentis*, were also found in the resident flora of the human oral cavity [15]. In addition, *Actinomyces* species colonize the upper respiratory tract, gastrointestinal tract and female genital tract [2,12,13,16]. In contrast, these microorganisms are not normally present on the surface of the skin, which is dominated by *Cutibacterium* (previously *Propionibacterium*) spp. and various staphylococci. This phenomenon may be due to the secretion of various molecules (fatty acids, antibacterial peptides) by the above mentioned microorganisms, detrimental to the survival of other species [17,18].

## 2. Epidemiology, Clinical Presentation

*Actinomyces* infections in general are considered to be rare (with a reported annual incidence of 1/300,000 persons), however, the incidence of anaerobic Gram-positive rods may be underestimated/underreported, as many laboratories still do not have the capabilities or interest for their full diagnostic workup [5,19]. Additionally, these microorganisms are slow-growing and have fastidious nutritional requirements, which further discourages some microbiology laboratories from their precise identification [20]. *Actinomyces* infections mainly occur among patients between 20 to 60 years of age, while the incidence is greater in males (male-to-female ratio: 3:1) with a peak between age 40–50 years [2,5,19,21]. Only circumstantial evidence was found why males are affected more frequently than females, mainly associated with environmental factors, while no physiological/biochemical/hormonal correlation was described. No racial, seasonal, geographical, or occupational predilections are associated with the increased occurrence of actinomycoses, however, before the 1970s, an increased prevalence was noted in rural areas, compared to people living in urban environments (the observed prevalence was 10:1; cervicofacial and cutaneous actinomycosis: presumably due to poor hygiene, neglected health status, and low socioeconomic status; pelvic: in females, due to extended (>5 years) use of intrauterine devices (IUDs) and inadequate knowledge level on sexual practices), especially in people working with farm animals [5,19]. Anatomically, *Actinomyces* infections may be divided to cervicofacial (including central nervous system), abdominal, thoracic (including lung), pelvic, and cutaneous infections (Table 2), with cervicofacial infections (also known as ‘lumpy jaw syndrome’) being the most prevalent type in the clinical practice [1,5,11,19,21,22,23,24,25,26,27,28,29,30,31]. In addition, *Actinomyces* species were also described in unusual clinical presentations, such as abscesses of the breasts, groin, perianal, periaural area, and axillae; infections of knee and hip prostheses; and pericarditis [1,5,11,19,21,22,23,24,25,26,27,28,29,30,31]. 

Thoracic (and lung) actinomycosis is primarily caused by the aspiration of oral *Actinomyces* from saliva (usually presenting with pyogenic infections in the chest wall, ribs and spine), abdominal infections arise from bowel perforation or increased intestinal permeability (resulting in appendicitis and colonic or diverticular infections), pelvic actinomycosis is linked to the extensive (i.e., long term) use of IUDs, while cerebral actinomycosis is primary caused by cervicofacial surgeries or penetrative head trauma [1,5,11,19,21,22,23,24,25,26,27,28,29,32]. Adequate and prompt detection of actinomycosis should be performed, as the associated mortality is between 0 and 28% [19]. Initiation of therapy in cervicofacial infections is also important for the containment of the infection, as in untreated cases, the pathogens may disseminate to distant organs—such as the brain, lungs, and gastrointestinal tract—resulting in concomitant infection [19,21,22,23,24,25,26,27,28,29]. In fact, hematogenous spread of bacteria from cervicofacial infections are the second main causes of brain and lung actinomycoses [32,33,34,35,36]. *Actinomyces* species are frequently isolated from the bloodstream of patients, following dental extractions (transient bacteremia) or due to increased permeability of oral mucosa [37,38]. This kind of *Actinomyces* bacteremia is transient and should be cleared from the bloodstream in individuals with a healthy immune system [39].

Cervicofacial actinomycosis (as well as *Actinomyces* infections in other anatomical regions) is characterized by a slowly progressing (indolent) infection, although in rare cases, a rapid and fulminant course of infection may also take place (especially in case of immunocompromised patients), which is caused by infiltration of *Actinomyces* species (present in the oral cavity) into damaged mucosal surfaces [11,21]. This mucosal injury may be due to inadequate dental hygiene, various medical interventions (mainly dental extractions or cervicofacial surgery), or trauma [1,5,11,19,21,22,23,24,25,26,27,28,29,32]. The general and disease-specific risk factors for developing cervicofacial actinomycosis is presented in Table 3. While the indolent/chronic form is a relatively painless process at first, patients with the acute/fulminant form may rapidly experience severe pain [11]. In these infections, chronic, granulomatous lesions develop, that over time become suppurative in character [5,19]. These lesions usually form multiple large abscesses (*cold abscesses*), which are connected by sinus tracts. Sinuses may express a typical, thick yellow exudate, containing the characteristic sulfur granules of *Actinomyces* spp. These granules, which are primarily yellow (but become dark brown later on, due to the deposition of calcium-phosphate) contain masses of filamentous organisms, bound together by the calcium salts and biofilm (a protein–polysaccharide complex) [5,19,40]. These granules macroscopically resemble grains of sand (0.1–1 mm in diameter) and they are important diagnostic markers of *Actinomyces* species [5,19]. The granulomatous lesions do not respect tissue borders, therefore in advanced stages (due to the infiltration of the muscles used in mastication and the jawbone; 10% of cases), severe pain, difficulties in chewing, trismus may also occur [11]. The affected tissues usually have a hard (some reports say woody) consistency, due to the corresponding fibrosis and scarring.

The disease is often characterized by non-specific febrile episodes, coughs, and sudden weight loss, while adenopathy is rarely observed. Laboratory findings may include leukocytosis, increased erythrocyte sedimentation rate (ESR) and C-reactive protein (CRP) levels [41,42]. Of note, the time period between the onset of the (non-specific) symptoms and the clinical diagnosis may be very long (6–12 months), which corresponds to erosion and tissue damage in the affected area. The unspecific nature of the symptoms further delays patients in seeking medical attention [43]. During the formulation of diagnosis, the clinical presentation, previous medical history of the patient should be considered, together with the microbiological findings; the growth of *Actinomyces* species does not definitively confirm and the absence of the pathogens in culture does not definitively rule out the disease [41,42,43].

The clinical diagnosis of actinomycosis may be difficult as the clinical presentation of the signs/symptoms can mimic other pathologies, such as solid tumors, active *Mycobacterium tuberculosis* infections, nocardiosis, fungal infections, infarctions (in the lungs), or other granulomatous diseases [5,21,44,45,46]. Although *Actinomyces* species are the primary cause of actinomycoses, there are usually multiple bacterial species present in these lesions: these pathogens include oral facultative pathogens, such as *Aggregatibacter actinomycetemcomitans*, *Propionibacterium propionicum, Streptococcus vidirans* group, Gram-positive anaerobic cocci (GPAC), and Gram-negative anaerobic rods [9,47,48]. If the physicians observe suppurative lesions in the submandibular or perimandibular regions, draining sinus tracts along the jawline and neck, the suspicion of actinomycosis should arise, especially in the case of relapsing infections [5,21,44,45,46].

*Actinomyces* species have been implicated in two other cervicofacial pathologies, that were previously considered non-infectious diseases: bisphosphonate osteonecrosis of the jaw and osteoradionecrosis [5,19,49,50,51,52]. In both cases, *Actinomyces* spp. and biofilm containing sulfur granules were detected in necrotic bone lesions, presumably further facilitating bone tissue damage. Radiation therapy is characterized by mucosal disruption and corticosteroid use, while bisphosphonate therapy in elderly patients is due to osteoporosis; both clinical situations usually correspond to patients with advanced age and severe underlying conditions, and these medical interventions may additionally facilitate the invasion of the jawbone by these pathogens (although the exact pathomechanism of these diseases is still not clear) [5,19,49,50,51,52].

## 3. Pathogenesis of the Infection

There is not much known about the virulence factors of anaerobic non-spore-forming Gram-positive bacteria [19]. Members of this group (including *Actinomyces* spp.) are considered to be low-grade pathogens, without possessing ‘classical’ virulence factors, such as exotoxins [13,14,18,53,54]. Consequently, these microorganisms can only cause disease if the normal mucosal barriers have been disrupted by exogenous or iatrogenic damage (e.g., surgical intervention, trauma, foreign bodies, concomitant infections), which explains why the mentioned predisposing factors are necessary for these infections to occur [14,18,53,54]. As *Actinomyces* spp. are abundantly present in the normal flora of humans (especially in the oral microbiome), these infections are mainly endogenous [5,14]. *Actinomycoses* infections of an exogenous source or interpersonal transmission were not been reported, apart from so-called “punch or knuckle” actinomycosis, corresponding to punching or biting injuries [55].

Due to their ability to cause chronic lesions, these microorganisms should have the ability to evade elimination by the immune system [3,41]. It is hypothesized that the formation of dense, interlinked chains of branched bacteria in sulfur granules should act as an inhibitor of phagocytic clearance; however, this phenomenon was not detected in an animal model, where bacteria were quickly phagocytosed by host defenses [40,56]. As *Actinomycosis* infections are usually polymicrobial, involving as many as 5–10 other bacterial species simultaneously, these multispecies (and sometimes multiphyla) communities are thought to contribute to the pathogenesis of these infections [57]. In polymicrobial infections, aerobic/facultative anaerobic bacteria enhance the survival of anaerobes by reducing the oxygen tension in the tissues, by the production of a capsule or toxins, damaging the mucosal barrier, enhancing the invasiveness of *Actinomyces* species [18,56]. In addition, the destruction of host tissues is beneficial for these microbial communities, providing nutrients and Fe^2+^ ions for all members of the consortia [40,56]. The presence of foreign bodies (associated with devitalized tissues) further increases the possibility of invasion [58]. Strong biofilm production has been described in case of *A. israelii*, *A. naeslundii*, and *A. viscosus*; the biofilm may have a role in the chronic nature of these infections and their recurrence, and protects against the diffusion of antibiotic molecules [19]. The role of several other commensal bacteria (e.g., streptococci, *Aggregatibacter actinomycetemcomitans*, *Eikenella corrodens*, *Haemophilus aprophilus*, and *P. propionicum*) has been noted in the inhibition of host defenses after a mucosal breach [9,48]. 

In addition, the relevance of *A. odontolyticus*, *A. oris*, *A. naeslundii*, and *A. viscosus* in the formation of dental plaques was described: these microorganisms are so-called “early colonizers” and they have the ability to bind statherin and proline-rich proteins in the saliva by their fimbriae, allowing for their binding onto the surface of teeth [19,33,59,60,61,62]. On these surfaces, they may also interact (co-aggregate) with other members of the dental plaque microorganisms (e.g., *Fusobacterium*, *Prevotella*, and *Veillonella* species). In the presence of fermentable carbohydrates, *Actinomyces* species also produce acid, leading to dental caries and tooth decay. The significance of these fimbriae is also pronounced in osteomyelitis, where they bind collagen and contribute to the pathogenesis of bone necrosis [63,64,65,66]. It has been described that *A. meyeri* has a great propensity for hematogenous dissemination; however, no differentiating virulence factor has been identified [22,35]. Similarly, several species (*A. naeslundii*, *A. odontolyticus*, *A. gerencseriae*, *A. neuii*, *A. turicensis*, and *A. radingae*) have been implicated in anatomically specific clinical syndromes, which may be mediated by some form of tissue tropism or specific virulence determinant [19,33,59,60,61,62]. 

## 4. Isolation and Identification of *Actinomyces* spp. from Clinical Specimen

**Figure 1:**
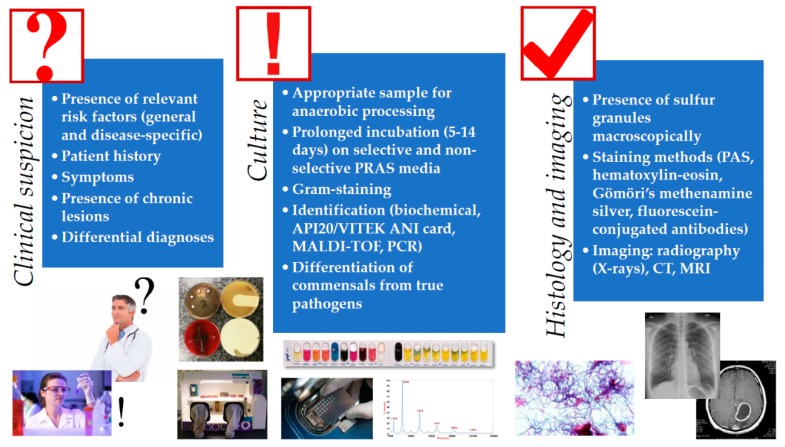
Schematic diagnostic algorithm for the clinical diagnosis of *Actinomyces* infections (for detailed explanation, see Section 2, Section 3 and Section 4).

Isolation and identification of *Actinomyces* spp. is a very important step in the diagnostic procedure of this disease and laboratory confirmation of these infections is often difficult [5]. The gold standard for the diagnosis of cervicofacial actinomycosis is culture, histological examination of a tissue sample, pus or abscess. Similarly to other anaerobic pathogens, the best clinical specimens for isolation are tissue biopsy samples, deep needle aspirates, joint fluids, pus, root canal exudates, aseptically collected peripheral blood and subgingival plaque (or bone, if osteomyelitis is suspected); while cutaneous or mucosal swabs, sputum, bronchial washing liquid (except for MiniBAL) and urine (except for suprapubic bladder aspirates) are not suitable for identification [10]. In addition, *Actinomyces* needs to be isolated from anatomical areas possessing rich commensal microbiota, therefore special care needs to be taken during sample collection (Figure 1) [67]. Depending on the infection site, *Actinomyces* may be co-isolated with normal commensals, such as members of the *Enterobacteriaceae*, *Staphylococcus* spp., *Streptococcus* spp., and *E. corrodens* among facultative anaerobes; and *A. actinomycetemcomitans, Bacteroides* spp., *Capnocytophaga* spp., *Prevotella* spp., and *Veilonella* spp. among anaerobes [18,67,68]. For this reason, isolation of *Actinomyces* from a sterile body site (or the isolation of a limited number of microorganisms) usually has clinical significance, while from other sample types, the isolation of these microorganisms can be considered as contamination (or their significance should be evaluated by the treating physician). If polymicrobial infections is suspected, obtaining pure cultures of all of the relevant organisms present should be attempted. Additionally, recent broad-spectrum antibiotics may also reduce microbial diversity, which may also be misleading [5,10].

The use of anaerobic transport techniques and pre-reduced anaerobically sterilized (PRAS) media is essential for successful recovery of these microorganisms [18]. Although these pathogens are more tolerant to atmospheric oxygen than most anaerobic bacteria, *Actinomyces* often fail to grow aerobically; therefore, the culture plates should be incubated in strict anaerobic condition (especially for their primary isolation [5,69]. This may be performed using anaerobic jars or boxes, with the help of GasPak™ sachets (convenient for smaller laboratories, but these should not be prematurely opened as this could result in an oxygen shock for anaerobic microorganisms) or anaerobic glove boxes (ideal for reference laboratories with large volume of samples, allows for manipulation and examination of samples in anaerobic atmosphere, expensive) [10,18,70]. Both enriched culture broth (brain–heart infusion) and non-selective and selective solid media (arginine–glycerine agar) should be used for the successful isolation of *Actinomyces* spp.; metronidazole may act a selective agent for most other anaerobes, while nalidixic acid should be used to eliminate facultative anaerobic Gram-negative bacteria [67,71]. *Actinomyces* spp. are fastidious, slow-growing microorganisms, bacterial colonies may appear after 3–7 days; however, this may take up to 14 days, therefore extended incubation times should be applied before the final microbiological report is released [72]. The communication between the clinician and microbiologist is imperative in these cases, as the physicians should notify the laboratory about the suspicion of cervicofacial actinomycosis [43]. In case of bone involvement, even longer incubation is recommended, due to the reduced growth properties of bacteria in this niche [43]. Although it is important to note that in around ~50% of cases, culture results are negative, especially if the samples were taken after the onset of antimicrobial therapy (which is frequent following dental procedures or cervicofacial surgery) [20]. Presumptive identification of these microorganisms is based on their morphology (clumping in liquid culture, white colonies with a domed surface or molar tooth-shaped morphology on solid media, which may become irregular after an extended incubation time), Gram-stain morphology, acid-fast negativity, indole-negativity, and metronidazole-resistance [8,67,73]. Additional (species-level) identification may be performed in tertiary-care centers or anaerobic reference laboratories, using biochemical testing methods (e.g., in-house biochemical panels, API 20A, VITEK 2 ANC card), gas-liquid chromatography, molecular methods (DNA–DNA hybridization, PCR, 16S rRNA gene sequencing, and fluorescence in situ hybridization), and MALDI-TOF MS [51,74,75,76,77,78]. Serology is of no relevance in the diagnosis of actinomycosis [5,21,41].

The use of MALDI-TOF MS for diagnostic purposes has revolutionized routine clinical microbiology, and this is especially true for the identification of anaerobic bacteria [79,80,81,82]. MALDI-TOF MS has allowed for the correct and precise identification of anaerobes in a clinically-relevant time frame, right after the appearance of the bacterial colonies on agar media, reducing the turnaround time for the processing of individual samples (compared to conventional, biochemical-based methods) [83,84]. Currently, two major MALDI-TOF MS systems are available: the Microflex MALDI-TOF MS (Bruker Daltonik GmbH, Bremen, Germany) and the VITEK MS MALDI-TOF (bioMérieux) [85,86]. Both of these instruments utilize a database of existing reference spectra (Biotyper for Bruker (Bruker Daltonik GmbH, Bremen, Germany) and Saramis for bioMérieux (bioMérieux, Marcy-l’Étoile, France)) [85]. Several studies have reported on the identification efficacy of *Actinomyces* using direct inoculation onto the MALDI-TOF stainless steel plate; however, on-plate extraction with 70% ethanol or formic acid or a complete extraction before the MALDI-TOF measurements has increased the rate of species-level identification for Gram-positive anaerobes [87]. However, there are currently no studies detailing on the clinical impact (i.e., benefit) of species-level identification of *Actinomyces* species, as the therapeutic approach for these infections is very similar, irrespective of the species of the pathogen [88,89].

The macroscopic and direct microscopic examination of pus collected from the suspected lesions is of critical importance in the diagnosis of actinomycosis [5]. As the colony counts of these microorganisms is usually low in pus, a large volume of sample or several samples should be collected and examined. Sulfur granules are suggestive of the presence of *Actinomyces* spp., while contamination may be suspected in the absence of these granules and a typical clinical syndrome, however, it is not specific enough for definitive diagnosis (they are absent in ~50% of samples, and similar granules may be observed in *N. asteroides/brasiliensis* and *Streptomyces madurae* infections) [19]. If possible, these sulfur granules from the sinuses or tissue biopsy should be crushed and examined microscopically after Gram-staining (or methylene blue), where Gram-positive, thin rods should be observed with a branching morphology. These bacterial cells may also be surrounded by inflammatory (polymorphonuclear leukocytes, PMN) cells, plasma cells and fibroblasts [1]. Histological examination of tissue samples is also an important cornerstone of diagnosis. Various staining methods, such as periodic acid-Schiff staining, hematoxylin-eosin, Gömöri’s methenamine silver stain, and fluorescein-conjugated specific antibodies may be used for this purpose [1]. Histological examination detects the characteristic granules and bacterial mycelia in ~75% of cases [19]. Cytology is the most sensitive method for the detection of *Actinomyces* spp. in suspected pelvic actinomycosis [26]. Although they may not be contributory to the establishment of positive diagnosis, imaging technologies may be useful in the differentiation of suppurative lesions caused by *Actinomyces* from malignancy [29,44,45,46,73,90,91,92,93]. Dental panoramic radiographs, computer tomography (CT), and magnetic resonance imaging (MRI) are also valuable in the evaluation of the extent of tissue involvement (whether bone tissue is involved) and size and location of the granulomatous lesion [29,44,45,46,73,90,91,92,93] (Figure 1).

## 5. Therapy of Actinomycoses

When considering the antimicrobial therapy of *Actinomyces* infections, clinicians should be aware of the antibiotics having anti-anaerobe activity [94]. The complete list of these antibiotics, the rationale behind their clinical efficacy and global resistance levels are beyond the scope of this review, for further reading, see another publication by the authors [18]. Anaerobes with a high oxygen tolerance due to the presence of various enzymes (i.e., *Actinobaculum, Actinomyces, Bifidobacterium, Lactobacillus, Propionibacterium*) or strictly anaerobic genera (e.g., *Atopobium, Eggerthella, Eubacterium*, and *Mobiluncus*) due to other mechanisms are intrinsically resistant to metronidazole, which is considered to be the therapy-of-choice for other anaerobic genera (e.g., *B. fragilis*, *Clostridium* species) [95,96]. Aminoglycosides have no activity against anaerobes in general (including *Actinomyces*), while fluoroquinolones (ciprofloxacin, levofloxacin, and moxifloxacin), sulfamethoxazole/trimethoprim are usually considered to be inactive [97,98]. Among β-lactam antibiotics, penicillinase-stable penicillins (such as oxacillin and cloxacillin), first generation cephalosporins (cephalexin) and aztreonam are also ineffective. Doxycycline could be considered as a viable therapeutic option (and there are several reports of clinical success with this drug); however, resistance levels in anaerobes globally are very high, discouraging the use of this agent in first-line therapy [18,32,99]. 

The first-line therapy of actinomycosis is high-dose therapy with intravenous penicillin G (12–24 million U/day for adults) or ampicillin for 2–6 weeks, which should be replaced in a sequential fashion (and if clinical improvement is observed) by penicillin V or amoxicillin *per os* for an extended period of time (6–12 months) to prevent relapse [1,5,19,100]. If it is clinically reasonable, intravenous agents may include more broad-spectrum agents, such as piperacillin-tazobactam, cefoxitin, ceftriaxone, and carbapenems. In the case of penicillin-hypersensitivity, other drugs should be considered: clindamycin (good penetration into abscesses), macrolides (erythromycin, clarithromycin, or azithromycin), doxycycline, tigecycline, and chloramphenicol [27,101,102,103]. In actinomycoses, the elimination of *Actinomyces* spp. is generally considered to be adequate for clinical cure, however in severe cases or for immunosuppressed patients, antibiotic therapy should target all isolated species [11]. If polymicrobial infection is suspected, a β-lactam/β-lactamase-inhibitor combination, carbapenems, aminoglycosides (e.g., for *Enterobacteriaceae*), or metronidazole (against other anaerobes) should also be administered in the therapeutic regimen [41]. However, the dogma of prolonged therapy in actinomycoses has been challenged by several reports, where shorter (1–4 week) therapeutic regimens were also reported to be successful [104,105,106] (Table 4). Especially in mild cases (i.e., no bone involvement) of cervicofacial and thoracic actinomycoses, satisfactory cure rates have been observed with short-term oral-only antibiotic therapy, coupled with proper surgical debridement, without relapses. Nevertheless, more clinical studies are required to warrant a change in current therapeutic guidelines, as this topic is still very controversial [104,105,106].

Resistance levels of anaerobic bacteria are generally considered to be predictable, and because of this, routine antimicrobial susceptibility testing is not indicated in *Actinomyces* spp., only for reference laboratories for surveillance purposes [107,108]. The gold standard method for antimicrobial susceptibility testing of anaerobes is the agar dilution method (recommended by the Clinical and Laboratory Standards Institute; CLSI), which is a very expensive and labor-intensive technique, recommended for anaerobic reference centers [109,110]. Drug resistance (excluding intrinsic resistance mechanisms) is not a relevant issue in *Actinomyces* species, as their susceptibility levels to penicillin, amoxicillin, piperacillin-tazobactam, ceftriaxone, and carbapenems is near 100%, although the use of broad-spectrum agents is discouraged if additional etiologies are not considered [48,107,108]. However, some minor differences between *Actinomyces* species have been observed: while these microorganisms do not produce β-lactamases, *bla*_TEM_-type β-lactamases were detected in *A. graevenitzii* and *A. europaeus*, conferring resistance to ceftriaxone and piperacillin-tazobactam [107,111]. Additionally, there have been reports of *Actinomyces* spp. with high minimum inhibitory concentrations for tetracycline and meropenem [19]. Despite prolonged therapy with large doses of antibiotics, recurrence actinomycoses is common; however, this problem is more pertaining to pharmacokinetics (i.e., poor tissue penetration of antibiotics in the inflamed tissue, inadequate blood supply, and production of biofilm), and presumably not related to drug resistance [107,108].

Although antibiotic therapy is the basis of *Actinomyces* infections, surgical interventions should also be considered [1,5,19]. In mild cases, surgery may have an adjunctive role in therapy (as debridement reduces the formation of scar tissue) and may reduce the duration and dose of antibiotic treatment required. However, in severe cases, drainage of the formed abscesses to relieve obstruction, excision of sinus tracts, fistulas, and necrotic tissues is required for clinical success. Imaging techniques may be used to monitor response to treatment [1,5,19,92]. Additionally, the presence of any malignancy can be also ruled out during surgical intervention. There are several reports on the therapeutic use of photodynamic therapy and laser therapy; however, the relevance of these techniques in clinical practice should be further evaluated [1,5,19,100]. For the prevention of cervicofacial actinomycoses, good oral hygiene practices, regular dental checkups (especially for patients with dental prostheses), and specific lifestyle choices (pharmacological control of diabetes, cessation of smoking, reduced alcohol intake) are recommended.

## 6. Conclusions

Due to its relatively rare occurrence, human *Actinomyces* infections are considered to be a neglected disease (the WHO does not maintain a current statistical database on the global prevalence of actinomycoses); however, due to an increase in the number of immunocompromised patients and the wider availability of dental procedures worldwide, the prevalence of this illness is thought to be increasing. Especially in the case of cervicofacial actinomycosis (the most prevalent form of the disease), clinicians—from primary care physicians to surgeons—dentists and clinical microbiologists should familiarize themselves with this condition to allow for its prevention (in the presence of relevant risk factors) appropriate diagnosis and therapy. The distinction between actinomycosis and other infectious (tuberculosis, nocardiosis, fungal infections) and non-infectious (tumors) pathologies should be the first diagnostic step, and all relevant diagnostic modalities should be utilized. Because culture results and the presence of the characteristic sulfur granules is not specific enough for definitive diagnosis, the underlying conditions and present symptoms of the patient should be consulted. Patients usually present with extensive tissue damage and severe symptoms, due to the slow progression and indolent nature of the disease. On a positive note, the response to the adequately long antimicrobial therapy is usually good, even in patients where extensive necrosis and tissue damage has already occurred. Continuous education of healthcare professionals and a high degree of suspicion is imperative for the apt management of *Actinomycosis* infections.

## 7. Literature Search

To formulate the present manuscript, a literature search was performed by the authors in the PubMed/MEDLINE, EMBASE, and Web of Science databases up to 30th of May, 2019, using the following search keys: “*Actinomyces* OR *actinomycosis* OR *actinomycoses* OR *lumpy jaw syndrome* OR *A. gerencseriae* OR *A. israelii* OR *A. meyeri* OR *A. odontolyticus*”. There were no restrictions on article type or language. Two independent authors (M.G. and G.T.) examined the search results and selected papers based on the suitability to be included in this review paper. After the selection of appropriate articles, the reference list of these papers was also screened for relevant papers in the topic. Additionally, in case of several sub-topics of the review (e.g., MALDI-TOF for the diagnostics of anaerobes), authors also used references from their personal collection, totaling in n = 111 references.

## Figures and Tables

**Table 1:** *Actinomyces* species implicated in human infections [1,3,5,7,8,10,11].

*A. bovis*	*A. graevenitzii*	*A. nasicola*	*A. radingae*
*A. cardiffiensis*	*A. hominis*	*A. neuii*	*A. timonensis*
*A. dentalis*	*A. hongkongiensis*	***A. odontolyticus***	*A. turicensis*
*A. europaeus*	***A. israelii***	*A. oris*	*A. urogenitalis*
*A. funkei*	*A. massiliensis*	*A. oricola*	*A. viscous*
*A. georgiae*	***A. meyeri***	*A. pyogenes*	*several novel geno-species*
***A. gerencseriae***	*A. naeslundii*	*A. radicidentis*	

Species in ***boldface*** represent >90% of species isolated from human infections; *A. odontolyticus* and *A. meyeri* are more frequently implicated in infections of the cervicofacial region, while *A. gerencseriae* and *A. israelii* are prevalent in all types of actinomycoses.

**Table 2:** Clinical manifestations of *Actinomyces* infections [1,5,11,19,21,22,23,24,25,26,27,28,29,30,31].

*Cervicofacial (including Central Nervous System (CNS))*	40–60%
*Affected areas:*	
Upper and lower mandibles	50%
Cheeks	10–15%
Chin	10–15%
Submaxillary ramus and angle, mandibular joints	5–10%
CNS (brain abscess, meningitis, meningoencephalitis, epidural abscess, subdural empyema)	5–10%
Tongue, sinuses, middle ear, larynx, lachrymal pathways, and thyroid gland	0–5%
*Abdominal*	20–30%
*Thoracic (incl. pulmonary)*	20–25%
*Pelvic*	3–5%
*Cutaneous*	3–5%

**Table 3:** General and disease-specific risk factors for cervicofacial actinomycosis [1,5,11,19,21,22,23,24,25,26,27,28,29,41,42,43].

General	Disease-Specific
Human Immunodeficiency Virus (HIV) infection or manifest AIDS	Erupting secondary teeth
Hematological malignancies or solid tumors	Poor dental hygiene
Organ transplantation (especially in case of the kidneys and lungs)	Dental caries
Use of monoclonal antibodies (e.g., anti-tumor necrosis-α-inhibitors, infliximab, etanercept)	Gingivitis
Cancer chemotherapy	Mucositis
Corticosteroid use	Dental extraction
Malnutrition	Introduction of dental implants
Diabetes	Cervicofacial surgery
Alcoholism	Traumatic injury
Smoking and/or inhalation of particles	Bisphosphonate therapy
Low socio-economic status	Radiation therapy
Seizure disorders	
Crohn’s disease	
Hereditary diseases (e.g., hereditary hemorrhagic telangiectasia, chronic granulomatous disease)	
Use of non-steroid anti-inflammatory drugs (NSAIDs)	

**Table 4:** Therapeutic considerations for *Actinomyces* infections.

**Antibiotic Therapy**	
**Should be Considered**	**Should NOT be Considered**
Penicillin G	Metronidazole *
Penicillin V	Aminoglycosides *
Ampicillin	Other β-lactam-β-lactamase-inhibitor combinations *
Amoxicillin	Penicillinase-stable penicillins
Piperacillin/tazobactam	First generation cephalosporins (cephalexin)
Second generation cephalosporins with anti-anaerobic activity (cefoxitin)	Aztreonam *
Third generation cephalosporins (ceftriaxone)	Sulfamethoxazole/trimethoprim *
Carbapenems	Fluoroquinolones *
Doxycycline	
Tigecycline	
Clindamycin	
Macrolides	
Chloramphenicol	
**Adjunctive Therapy**	
Surgical debridement, drainage	
Photodynamic therapy	
Laser therapy	

* Should be reconsidered in case of polymicrobial infections (e.g., *Enterobacteriaceae*, other anaerobes).

## Abbreviations

AIDS	acquired immunodeficiency syndrome
CNS	central nervous system
CRP	C-reactive protein
CT	computer tomography
DNA	deoxyribonucleic acid
ESR	erythrocyte sedimentation rate
G+C	guanine-cytosine content
GPAC	Gram-positive anaerobic cocci
HIV	human immunodeficiency virus
HMP	Human Microbiome Project
IUD	intrauterine device
MALDI-TOF MS	matrix-assisted laser desorption/ionization time-of flight mass spectrometry
MRI	magnetic resonance imaging
NSAID	non-steroidal anti-inflammatory drugs
NGS	next-generation sequencing
NIH	National Institutes of Health
PCR	polymerase chain reaction
PMN	polymorphonuclear neutrophils
PRAS	pre-reduced anaerobically sterilized
WHO	World Health Organization

## References

[1] Könönen E., Wade W.G. (2015). *Actinomyces* and related organisms in human infections. Clin. Microbiol. Rev..

[2] Finegold S.M., Mandell G.L., Bennett J.E., Dolin R. (2000). Anaerobic infections: General concepts. Principles and Practice of Infectious Diseases.

[3] Zhao K., Li W., Kang C., Du L., Huang T., Zhang X., Wu M., Yue B. (2014). Phylogenomics and evolutionary dynamics of the family *Actinomycetaceae*. Genome Biol. Evol..

[4] Holmberg K., Nord C.E. (1975). Numerical taxonomy and laboratory identification of *Actinomyces* and *Arachnia* and some related bacteria. J. Gen. Microbiol..

[5] Boyanova L., Kolarov R., Mateva L., Markovska R., Mitov I. (2015). *Actinomycosis*: A frequently forgotten disease. Future Microbiol..

[6] Murray P.R., Baron E.J., Jorgensen J.H., Landry M.L., Pfaller M.A. (2007). Manual of Clinical Microbiology.

[7] Sullivan D.C., Chapman S.W. (2010). Bacteria that masquerade as fungi: Actinomycosis/nocardia. Proc. Am. Thorac. Soc..

[8] Thukral R., Shrivastav K., Mathur V., Barodiya A., Shrivastav S. (2017). *Actinomyces*: A deceptive infection of oral cavity. J. Korean Assoc. Oral Maxillofac. Surg..

[9] Bowden G.H.W., Baron S. (1996). *Actinomyces*, *Propionibacterium propionicus*, and *Streptomyces*. Medical Microbiology.

[10] Nagy E., Boyanova L., Justesen U.S. (2018). How to isolate, identify and determine antimicrobial susceptibility of anaerobic bacteria in routine laboratories. Clin. Microbiol. Infect..

[11] Pulverer G., Schütt-Gerowitt H., Schaal K.P. (2003). Human cervicofacial actinomycoses: Microbiological data for 1997 cases. Clin. Infect. Dis..

[12] Evaldson G., Heimdahl A., Kager L., Nord C.E. (1982). The normal human anaerobic microflora. Scand. J. Infect. Dis. Suppl..

[13] Gajdács M., Urbán E. (2019). Epidemiology and species distribution of anaerobic Gram-negative cocci: A 10-year retrospective survey (2008–2017). Acta Pharm. Hung..

[14] Hall V. (2008). *Actinomyces*—Gathering evidence of human colonization and infection. Anaerobe.

[15] Turnbaugh P.J., Ley R.E., Hamady M., Fraser-Liggett C., Knight R., Gordon J.I. (2007). The human microbiome project: Exploring the microbial part of ourselves in a changing world. Nature.

[16] Finegold S.M. (1977). Anaerobic Bacteria in Human Disease.

[17] Bitschar K., Sauer B., Focken J., Dehmer H., Moos S., Konnerth M., Schilling N.A., Grond S., Kalbacher H., Kurschus F.C. (2019). Lugdunin amplifies innate immune responses in the skin in synergy with host- and microbiota-derived factors. Nat. Commun..

[18] Gajdács M., Spengler G., Urbán E. (2017). Identification and Antimicrobial Susceptibility Testing of Anaerobic Bacteria: Rubik’s Cube of Clinical Microbiology?. Antibiotics.

[19] Vaulor F., Sénéchal A., Dupieux C., Karsenty J., Lustig S., Breton P., Gleizal A., Boussel L., Laurent F., Braun E. (2014). Actinomycosis: Etiology, clinical features, diagnosis, treatment, and management. Infect. Drug Res..

[20] Leber A.L. (2016). Clinical Microbiology Procedures Handbook.

[21] Oostman O., Smego R.A. (2005). Cervicofacial Actinomycosis: Diagnosis and Management. Curr. Infect. Dis. Rep..

[22] Clérigo V., Fernandes L., Feliciano A., Carvalho L. (2017). A rare case of *Actinomyces meyeri* empyema: Still a challenging entity to manage. Respir. Med. Case Rep..

[23] Crossman T., Herold J. (2009). Actinomycosis of the maxilla—A case report of a rare oral infection presenting in general dental practice. Br. Dent. J..

[24] Garner J.P., Macdonald M., Kumar P.K. (2007). Abdominal actinomycosis. Int. J. Surg..

[25] Grzywa-Celińska A., Emeryk-Maksymiuk J., Szmygin-Milanowska K., Czekajska-Chehab E., Milanowski J. (2017). Pulmonary actinomycosis—The great imitator. Ann. Agric. Environ. Med..

[26] Matsuda K., Nakajima H., Khan K.N., Tanigawa T., Hamaguchi D., Kitajima M., Hiraki K., Moriyama S., Masuzaki H. (2012). Preoperative diagnosis of pelvic actinomycosis by clinical cytology. Int. J. Womens Health.

[27] Kim S.R., Jung L.Y., Oh I.-J., Kim Y.-C., Shin K.-C., Lee M.K., Yang S.-H., Park H.S., Kim M.-K., Kwak J.Y. (2013). Pulmonary actinomycosis during the first decade of 21st century: Cases of 94 patients. BMC Infect. Dis..

[28] Palmitessa V., Cuppone R., Monno R., Fumarola L., Lippolis A. (2019). A case report of esophageal actinomycosis in an immunocompetent patient and review of the literature. New Microbiol..

[29] Reichenbach J., Lopatin U., Mahlaoui N., Beovic B., Siler U., Zbinden R., Seger R.A., Galmiche L., Brousse N., Kayal S. (2009). *Actinomyces* in Chronic Granulomatous Disease: An Emerging and Unanticipated Pathogen. Clin. Infect. Dis..

[30] Li J., Li Y., Zhou Y., Wang C., Wu B., Wan J. (2018). *Actinomyces* and Alimentary Tract Diseases: A Review of Its Biological Functions and Pathology. BioMed Res. Int..

[31] Slutzker A.D., Claypool W.D. (1989). Pericardial actinomycosis with cardiac tamponade from a contiguous thoracic lesion. Thorax.

[32] Gajdács M., Urbán E. (2019). The relevance of anaerobic bacteria in brain abscesses: A ten-year retrospective analysis (2008–2017). Infect. Dis..

[33] Clancy U., Ronayne A., Prentice M.B., Jackson A. (2015). *Actinomyces meyeri* brain abscess following dental extraction. BMJ Case Rep..

[34] Hwang C.S., Lee H., Hong M.P., Kim J.H., Kim K.-S. (2018). Brain abscess caused by chronic invasive actinomycosis in the nasopharynx. Medicine.

[35] Vazquez Guillamet L.J., Malinis M.F., Meyer J.P. (2017). Emerging role of *Actinomyces meyeri* in brain abscesses: A case report and literature review. IDCases.

[36] Fan G., Gu J., He S., Cai X. (2016). Comprehensive management of cervical epidural spinal abscess followed by brain abscesses: A life-threatening and tortuous case. Int. J. Clin. Exp. Med..

[37] Benítez-Páez A., Álvarez M., Belda-Ferre P., Rubido S., Mira A., Tomás I. (2013). Detection of Transient Bacteraemia following Dental Extractions by 16S rDNA Pyrosequencing: A Pilot Study. PLoS ONE.

[38] Lockhart P.B., Brennan M.T., Thornhill M., Michalowicz B.S., Noll J., Bahrani-Mougeot F.K., Sasser H.C. (2009). Poor oral hygiene as a risk factor for infective endocarditis-related bacteremia. J. Am. Dent. Assoc..

[39] Lockhart P.B., Brennan M.T., Sasser H.C., Fox P.C., Paster B.J., Bahrani-Mougeot F.K. (2008). Bacteremia Associated with Tooth Brushing and Dental Extraction. Circulation.

[40] Nair P.N.R., Brundin M., Sundqvist G., Sjögren U. (2008). Building biofilms in vital host tissues: A survival strategy of *Actinomyces radicidentis*. Oral Surg. Oral Med. Oral Pathol. Oral Radiol. Endod..

[41] Wong V.K., Turmezei T.D., Weston V.C. (2011). Actinomycosis. BMJ.

[42] Hansen J.M., Fjeldsøe-Nielsen H., Sulim S., Kemp M., Christensen J.J. (2009). *Actinomyces* species: A Danish Survey on Human Infections and Microbiological Characteristics. Open Microbiol. J..

[43] Bonnefond S., Catroux M., Melenotte C., Karkowski L., Rolland L., Trouillier S., Raffray L. (2016). Clinical features of actinomycosis. Medicine.

[44] Bulut G., Bayram Y., Bulut M.D., Garça M.F., Bayram İ. (2017). Mandibular Actinomyces Infection Mimicking a Malignancy: Case Report. Turk. Patol. Derg..

[45] Ji W., Kwak J.M., Kim J., Kim S.H. (2014). Actinomycosis of the rectum mimicking a malignant neoplasm. ANZ J. Surg..

[46] Gliga S., Devaux M., Gosset-Woimant M., Mompoint D., Perrone C., Davido B. (2014). *Actinomyces graevenitzii* pulmonary abscess mimicking tuberculosis in a healthy young man. Can. Resp. J..

[47] Murphy E.C., Frick I.M. (2013). Gram-positive anaerobic cocci—Commensals and opportunistic pathogens. FEMS Microbiol. Rev..

[48] Tanaka-Bandoh K., Watanabe K., Kato N., Ueno K. (1997). Susceptibilities of *Actinomyces* species and *Propionibacterium propionicus* to antimicrobial agents. Clin. Infect. Dis..

[49] Hansen T., Kunkel M., Kirkpatrick C.J., Weber A. (2006). *Actinomyces* in infected osteoradionecrosis—Underestimated?. Hum. Pathol..

[50] Hansen T., Kunkel M., Springer E., Walter C., Weber A., Siegel E., Kirkpatrick C.J. (2007). Actinomycosis of the jaws—Histopathological study of 45 patients shows significant involvement in bisphosphonate-associated osteonecrosis and infected osteoradionecrosis. Virchows Arch..

[51] Støre G., Eribe E.R.K., Olsen I. (2005). DNA-DNA hybridization demonstrates multiple bacteria in osteoradionecrosis. Int. J. Oral Maxillofac Surg..

[52] Gallay L., Bodard A.-G., Chidiac C., Ferry T. (2013). Bilateral bisphosphonate-related osteonecrosis of the jaw with left chronic infection in an 82-year-old woman. BMJ Case Rep..

[53] Brook I. (2010). The role of anaerobic bacteria in bacteremia. Anaerobe.

[54] Hecht D.W. (2006). Anaerobes: Antibiotic resistance, clinical significance, and the role of susceptibility testing. Anaerobe.

[55] Blinkhorn R.J., Strimbu V., Effron D., Spagnuolo P.J. (1988). “Punch” actinomycosis causing osteomyelitis of the hand. Arch. Intern. Med..

[56] Nair P.N.R., Sundqvist G., Sjögren U. (2008). Experimental evidence supports the abscess theory of development of radicular cysts. Oral Surg. Oral Med. Oral Pathol. Oral Radiol. Endod..

[57] Gajdács M. (2019). The Concept of an Ideal Antibiotic: Implications for Drug Design. Molecules.

[58] Sarkonen N., Könönen E., Eerola E., Könönen M., Jousimies-Somer H., Laine P. (2005). Characterization of *Actinomyces* species isolated from failed dental implant fixtures. Anaerobe.

[59] Rôças I.N., Siqueira J.F. (2012). Antibiotic resistance genes in anaerobic bacteria isolated from primary dental root canal infections. Anaerobe.

[60] Marsh P.D. (2006). Dental plaque as a biofilm and a microbial community—Implications for health and disease. BMC Oral Health.

[61] Loesche W.J., Baron S. (1996). Microbiology of dental decay and periodontal disease. Medical Microbiology.

[62] Lovegrove J.M. (2004). Dental plaque revisited: Bacteria associated with periodontal disease. J. N. Z. Soc. Periodontol..

[63] Pizzo K., Arnold C., Wispelwey B. (2017). *Actinomyces neuii* Causing Vertebral Osteomyelitis. Am. J. Med. Sci..

[64] Shen J.Y., Futran N.D., Sardesai M.G. (2017). Craniofacial *Actinomyces* osteomyelitis evolving from sinusitis. Radiol. Case Rep..

[65] Lewis R.P., Sutter V.L., Finegold S.M. (1978). Bone infections involving anaerobic bacteria. Medicine.

[66] Sato T., Watanabe K., Kumada H., Toyama T., Tani-Ishii N., Hamada N. (2012). Peptidoglycan of *Actinomyces naeslundii* induces inflammatory cytokine production and stimulates osteoclastogenesis in alveolar bone resorption. Arch. Oral Biol..

[67] Jousimies-Somer H., Summanen P., Citron D.M., Baron E.J., Wexler H.M., Finegold S.M., Jousimies-Somer H., Summanen P., Citron D.M., Baron E.J., Wexler H.M., Finegold S.M. (2003). KTL Anaerobic Bacteriology Manual.

[68] Garg R., Kaistha N., Gupta V., Chander J. (2014). Isolation, Identification and Antimicrobial Susceptibility of Anaerobic Bacteria: A Study Re-emphasizing Its Role. J. Clin. Diag. Res..

[69] Morris R.L., Schmidt T.M. (2013). Shallow breathing: Bacterial life at low O_2_. Nat. Rev. Microbiol..

[70] Gajdács M. (2019). Anaerobes and laboratory automation: Like oil and water?. Anaerobe.

[71] Zimbro M.J., Power D.A., Miller S.M., Wilson G.E., Johnson J.A. (2009). Manual of Microbiological Culture Media.

[72] Gajdács M., Paulik E., Szabó A. (2018). The opinions of community pharmacists related to antibiotic use and resistance. Acta Pharm. Hung..

[73] Kalfas S., Edwardsson S. (1990). Identification procedures for oral *Actinomyces* species. Oral Microbiol. Immunol..

[74] Jamal W.Y., Shahin M., Rotimi V.O. (2013). Comparison of two matrix-assisted laser desorption/ionization-time of flight (MALDI-TOF) mass spectrometry methods and API 20AN for identification of clinically relevant anaerobic bacteria. J. Med. Microbiol..

[75] Sondag J.E., Ali M., Murray P.R. (1980). Rapid presumptive identification of anaerobes in blood cultures by gas-liquid chromatography. J. Clin. Microbiol..

[76] Schreckenberger P.C., Blazevic D.J. (1974). Rapid methods for biochemical testing of anaerobic bacteria. Appl. Microbiol..

[77] Nagy E., Becker S., Kostrzewa M., Barta N., Urban E. (2012). The value of MALDI-TOF MS for the identification of clinically relevant anaerobic bacteria in routine laboratories. J. Med. Microbiol..

[78] Kuyama K., Fukui K., Ochiai E., Maruyama S., Iwadate K., Saku T., Yamamoto H. (2013). Identification of the actinomycete 16S ribosomal RNA gene by polymerase chain reaction in oral inflammatory lesions. Oral Surg. Oral Med. Oral Pathol. Oral Radiol..

[79] Nagy E., Maier T., Urban E., Terhes G., Kostrzewa M., ESCMID Study Group on Antimicrobial Resistance in Anaerobic Bacteria (2009). Species identification of clinical isolates of Bacteroides by matrix-assisted laser-desorption/ionization time-of-flight mass spectrometry. Clin. Microbiol. Infect..

[80] Krishnamurthy T., Ross P.L., Rajamani U. (1996). Detection of pathogenic and non-pathogenic bacteria by matrix-assisted laser desorption/ionization time-of-flight mass spectrometry. Rapid Commun. Mass Spectr..

[81] Croxatto A., Prod’hom G., Greub G. (2012). Applications of MALDI-TOF mass spectrometry in clinical diagnostic microbiology. FEMS Microbiol. Rev..

[82] Seng P., Drancourt M., Gouriet F., La Scola B., Fournier P.-E., Rolain J.M., Raoult D. (2009). Ongoing revolution in bacteriology: Routine identification of bacteria by matrix-assisted laser desorption ionization time-of-flight mass spectrometry. Clin. Infect. Dis..

[83] Patel R. (2015). MALDI-TOF MS for the diagnosis of infectious diseases. Clin. Chem..

[84] Shannon S., Kronemann D., Patel R., Schuetz A.N. (2018). Routine use of MALDI-TOF MS for anaerobic bacterial identification in clinical microbiology. Anaerobe.

[85] Veloo A.C., Knoester M., Degener J.E., Kuijper E.J. (2011). Comparison of two matrix-assisted laser desorption ionisation-time of flight mass spectrometry methods for the identification of clinically relevant anaerobic bacteria. Clin. Microbiol. Infect..

[86] Lynch T., Gregson D., Church D.L. (2016). Species-Level Identification of Actinomyces Isolates Causing Invasive Infections: Multiyear Comparison of Vitek MS (Matrix-Assisted Laser Desorption Ionization-Time of Flight Mass Spectrometry) to Partial Sequencing of the 16S rRNA Gene. J. Clin. Microbiol..

[87] Veloo A.C., Erhard M., Welker M., Welling G.W., Degener J.E. (2011). Identification of Gram-positive anaerobic cocci by MALDI-TOF mass spectrometry. Syst. Appl. Microbiol..

[88] Ng L.S.Y., Sim J.H.C., Eng L.C., Menon S., Tan T.Y. (2012). Comparison of phenotypic methods and matrix-assisted laser desorption ionisation time-of-flight mass spectrometry for the identification of aero-tolerant *Actinomyces* spp. isolated from soft-tissue infections. Eur. J. Clin. Microbiol. Infect. Dis..

[89] Fong P., Francis M.J., Hamblin J.F., Korman T.M., Graham M. (2018). Identification and diversity of *Actinomyces* species in a clinical microbiology laboratory in the MALDI-TOF MS era. Anaerobe.

[90] Heo S.H., Shin S.S., Kim J.W., Lim H.S., Seon H.J., Jung S.-I., Jeong Y.Y., Kang H.K. (2014). Imaging of actinomycosis in various organs: A comprehensive review. Radiographics.

[91] Qiu L., Lan L., Feng Y., Huang Z., Chen Y. (2015). Pulmonary Actinomycosis Imitating Lung Cancer on (18)F-FDG PET/CT: A Case Report and Literature Review. Korean J. Radiol..

[92] Sasaki Y., Kaneda T., Uyeda J.W., Okada H., Sekiya K., Suemitsu M., Sakai O. (2014). Actinomycosis in the Mandible: CT and MR Findings. Am. J. Neuroradiol..

[93] Soneja M., Batra A., Vikram N.K., Ahuja A., Mohan A., Sood R. (2012). Actinomycosis and nocardiosis co-infection in chronic granulomatous disease. J. Assoc. Physicians India.

[94] Nagy E. (2010). Anaerobic Infections Update on Treatment Considerations. Drugs.

[95] Shinn D.L.S. (1962). Metronidazole in acute ulcerative gingivitis. Lancet.

[96] Alauzet C., Lozniewski A., Marchandin H. (2019). Metronidazole resistance and nim genes in anaerobes: A review. Anaerobe.

[97] Bryan L.E., Kowand S.K., Van Den Elzen H.M. (1979). Mechanism of aminoglycoside antibiotic resistance in anaerobic bacteria: Clostridium perfringens and Bacteroides fragilis. Antimicrob. Agents Chemother..

[98] Stein G.E., Goldstein E.J.C. (2006). Fluoroquinolones and anaerobes. Clin. Infect. Dis..

[99] Nagy E., Urbán E., Nord C.E., ESCMID Study Group on Antimicrobial Resistance in Anaerobic Bacteria (2011). Antimicrobial susceptibility of Bacteroides fragilis group isolates in Europe: 20 years of experience. Clin. Microbiol. Infect..

[100] Gajdács M. (2019). Intravenous or oral antibiotic therapy: Sophie’s choice?. Gen. Int. Med. Clin. Innov..

[101] Martin M.V. (1985). Antibiotic treatment of cervicofacial actinomycosis for patients allergic to penicillin: A clinical and in vitro study. Br. J. Oral Maxillofac. Surg..

[102] Moghimi M., Salentijn E., Debets-Ossenkop Y., Karagozoglu K.H., Forouzanfar T. (2013). Treatment of cervicofacial actinomycosis: A report of 19 cases and review of literature. Med. Oral Patol. Oral Cir. Bucal.

[103] Kolditz M., Bickhardt J., Matthiessen W., Holotiuk O., Höffken G., Koschel D. (2009). Medical management of pulmonary actinomycosis: Data from 49 consecutive cases. J. Antimicrob. Chemother..

[104] Atad J., Hallak M., Sharon A., Kitzes R., Kelner Y., Abramovici H. (1999). Pelvic actinomycosis: Is long term antibiotic therapy necessary?. J. Reprod. Med..

[105] Sudhakar S.S., Ross J.J. (2004). Short-term treatment of actinomycosis: Two cases and a review. Clin. Infect. Dis..

[106] Shah K.M., Karagir A., Kanitkar S., Koppikar R. (2013). An atypical form of cervicofacial actinomycosis treated with short but intensive antibiotic regimen. BMJ Case Rep..

[107] Barberis C., Budia M., Palombarani S., Rodriguez C.H., Ramírez M.S., Arias B., Bonofiglio L., Famiglietti A., Mollerach M., Almuzara M. (2017). Antimicrobial susceptibility of clinical isolates of *Actinomyces* and related genera reveals an unusual clindamycin resistance among *Actinomyces* urogenitalis strains. J. Glob. Antimicrob. Resist..

[108] Smith A.J., Hall V., Thakker B., Gemmell C.G. (2005). Antimicrobial susceptibility testing of *Actinomyces* species with 12 antimicrobial agents. J. Antimicrob. Chemother..

[109] Wexler H.M., Molitoris E., Murray P.R., Washington J., Zabransky R.J., Edelstein P.H., Finegold S.M. (1996). Comparison of spiral gradient endpoint and agar dilution methods for susceptibility testing of anaerobic bacteria: A multilaboratory collaborative evaluation. J. Clin. Microbiol..

[110] Clinical and Laboratory Standards Institute (CLSI) https://clsi.org/standards/products/microbiology/.

[111] Tietz A., Aldridge K.E., Figueroa J.E. (2005). Disseminated Coinfection with *Actinomyces graevenitzii* and Mycobacterium tuberculosis: Case Report and Review of the Literature. J. Clin. Microbiol..

